# The protective effects of Arctiin in asthma by attenuating airway inflammation and inhibiting p38/NF-κB signaling

**DOI:** 10.18632/aging.205584

**Published:** 2024-03-27

**Authors:** Lang Yuan, Chao Sun

**Affiliations:** 1 Department of Respiratory Medicine, Children's Hospital of Shanghai, Shanghai Jiaotong University, Shanghai 200062, China

**Keywords:** asthma, Arctiin, inflammation, oxidative stress, NF-κB

## Abstract

Asthma is a common chronic inflammatory disease of the airways, which affects millions of people worldwide. Arctiin, a bioactive molecule derived from the traditional Chinese Burdock, has not been previously reported for its effects on asthma in infants. In this study, an asthma model was established in mice by stimulation with ovalbumin (OVA). Bronchoalveolar lavage (BALF) was collected from OVA-challenged mice and the cells were counted. Lung tissue was harvested for hematoxylin-eosin (HE) staining and measurement of Wet/Dry weight ratios. The expressions of proteins were detected using enzyme-linked immunosorbent assay (ELISA) and Western blots. The superoxide dismutase (SOD) activity in lung tissue was measured using a commercial kit. We found that Arctiin had beneficial effects on asthma treatment. Firstly, it attenuated OVA-challenged lung pathological alterations. Secondly, it ameliorated pro-inflammatory response by reducing the number of inflammatory cells and mitigating the imbalance of Th1/Th2 factors in the bronchoalveolar lavage (BALF) of OVA-challenged mice. Importantly, Arctiin ameliorated OVA-induced lung tissue impairment and improved lung function. Additionally, we observed that oxidative stress (OS) in the pulmonary tissue of OVA-challenged mice was ameliorated by Arctiin. Mechanistically, Arctiin prevented OVA-induced activation of p38 and nuclear factor-κB (NF-κB). Based on these findings, we conclude that Arctiin might serve as a promising agent for the treatment of asthma.

## INTRODUCTION

Asthma is an important disease that affects human health, characterized by chronic airway inflammation, reversible airflow limitation, and bronchial hyperresponsiveness [1]. According to statistics from the World Health Organization (WHO), approximately 300 million patients worldwide suffer from asthma, and at least 250,000 people die from it annually, imposing a heavy burden on the social economy. Over the past 50 years, with the rise of industrialization and urbanization, the global prevalence of asthma has gradually increased. In China alone, adult asthma prevalence has reached 4.2% [2]. Patients with acute exacerbation of asthma are typically diagnosed with increased inflammation, airway mucus hypersecretion, and airway hyperresponsiveness (AHR). Multiple risk factors contribute to asthma exacerbations, such as allergens, respiratory infections, smoking, and air pollution. The cells recruited in the asthmatic immune-inflammatory cascade generate reactive oxygen species (ROS). This process enhances the production of oxidizing products, thereby activating redox-responsive intracellular signaling mechanisms [3]. Acute asthma attacks are heterogeneous due to the complex network of innate and adaptive host immune responses involved. The network of multiple molecules that elicit abnormal immune responses in the lungs during an acute asthma attack remains poorly understood [4]. Current treatment options mainly consist of glucocorticoids and B2 receptor agonists which are effective for stable asthma but have limited effects on acute asthma attacks. Therefore, exploring the factors affecting acute asthma attacks holds a very profound significance in exploring targets for asthma treatment and improving the life quality of patients [5]. The hallmark of allergen-induced asthma exacerbations is eosinophil infiltration primarily regulated by the type 2 immune response. In many individuals with asthma, the presence of Th2 cells or ILC2s that produce IL-4, IL-5, and IL-13 drives chronic airway inflammation. Some patients with Type 2-high asthma show persistent Th2-induced airway inflammation [6], while inhibition of Th2 cells through high-dose inhaled glucocorticoids reduces antibodies against type 2 cytokines and eosinophils, however, its therapeutic effects on asthma symptoms remain limited [7]. Furthermore, asthma is associated with respiratory viral infections and immune responses regulated by Th1 or Thl7 cells [8–10]. Other immune cells associated with respiratory antiviral immune responses, including macrophages, neutrophils, and natural killer (NK) cells, also participate in the development of asthma [11]. Regulating the balance of immune cells might be a promising strategy for treating asthma.

**Figure 1:**
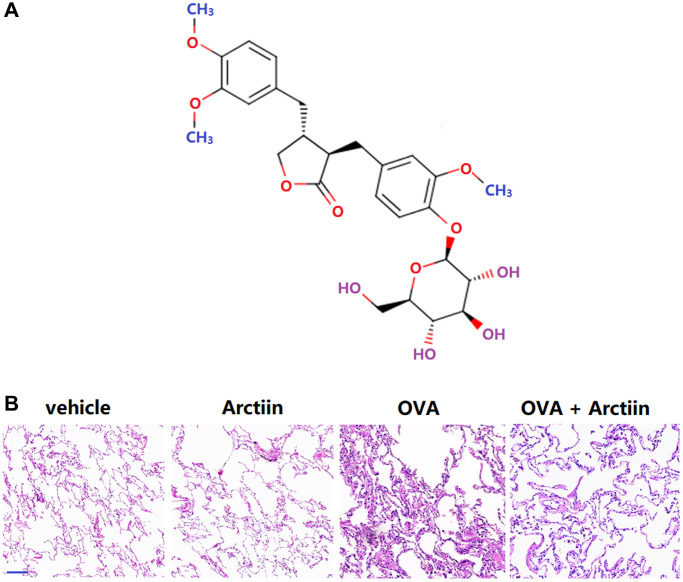
**Arctiin attenuated ovalbumin (OVA)-challenged lung pathological alterations.** C57BL/6 mice were divided into four groups: vehicle, Arctiin, OVA, and OVA+ Arctiin (10 mg/kg). (**A**) Molecular structure of Arctiin; (**B**) Lung histological changes were assessed using haematoxylin and eosin (HE) staining. Scale bar, 300 μm.

*Arctium lappa L* root is the dry and mature fruit of *Arctium lappa L*. in *Asteraceae*, which is commonly used in the clinical treatment of upper respiratory tract infection in traditional Chinese medicine [12]. Arctiin (Figure 1A) is a lignan compound extracted from roots of *Arctium lappa L* by alcohol and is one of the main active ingredients in *Arctium lappa L* roots. Modern pharmacological studies have shown that Arctiin promotes glucose uptake in tissues, inhibits α-glucosidase, increases insulin sensitivity, and achieves hypoglycemic effects. It is reported that Arctiin possesses anti-inflammatory, anti-viral, liver protection, and improvement of endothelial cell damage properties. Previous studies have attributed the anti-inflammatory effect of Arctiin to its potent *in vitro* and *in vivo* modulating effects on several crucial cytokines, including tumor necrosis factor-α (TNF-α), interleukin-6 (IL-6), interleukin-1β (IL-1β), interferon-γ (IFN-γ), and interleukin-10 (IL-10) [13–16]. However, whether Arctiin possesses a therapeutic effect on asthma remains unclear. Therefore, we hypothesize that Arctiin may exert protective effects on asthma by attenuating airway inflammation. Our study was designed to elucidate the underlying mechanism.

## MATERIALS AND METHODS

### Animal experiments

Forty-eight C57BL/6 mice, aged 7-8 weeks, and weighing about 30 grams (Well-bio, Changsha, China) were divided into four groups: vehicle, Arctiin, OVA, and OVA+ Arctiin. In the OVA group, mice were intraperitoneally injected with a sensitization solution (0.2 mL/mouse), containing 50 μg of OVA and 1 mg of aluminum hydroxide gel on the first day. On the 14th day, the mice were intraperitoneally injected with a sensitization solution (0.2 mL/mouse), containing 50 μg of OVA. Mice were made to inhale a 5% concentration of OVA for a duration of 20 minutes each time. The vehicle group received an equivalent volume of PBS buffer instead. In both the Arctiin and OVA+ Arctiin groups, normal mice and OVA-stimulated mice respectively were administered with a dose of 10 mg/kg Arctiin [17]. This study was approved by the animal care committee of The Children’s Hospital of Shanghai, Shanghai Jiaotong University.

### Hematoxylin-eosin (HE) staining

The collected lung tissues were rinsed with water for 2 h. After dehydration using different concentrations of ethanol solution, the tissues were further dehydrated with xylene until they were transparent, followed by embedding for 1 hour and sliced. After roasting, dewaxing, and hydration, the sections were immersed in water and stained by hematoxylin aqueous reagent for 2–3 minutes. Following differentiation using hydrochloric acid ethanol, the sections were stained with the blue-returning reagent finally, after being stained with eosin for 180 seconds, pictures were obtained using an inverted microscope (Leica, Germany) [18].

### Collection of BALF and cell counting

The chest of the mouse was opened, and the right lung bronchus was lapped with a thin line. The lavage needle was inserted into the trachea and connected to a syringe, with another thin line lapped around it to prevent falling off and air leakage. 0.3 mL PBS solution was drawn into the left lung through the trachea, and the recovered liquid was collected in the EP tube and repeated 3 times, with the recovery rate >80% as qualified. BALF was then centrifuged at 1500 r/min for 10 min at 4°C, and its supernatant was transferred to a new 1.5 mL EP tube and stored at −20°C for the determination of cytokines. Meanwhile, sediment from the bottom was re-suspended in a 20 μL Phosphate Buffered Saline (PBS) solution and mixed, then 2 μL was then applied onto the slides that were evenly spread out, dried, and stained with Diff-Quik Stain. Then, the slides were observed and photographed under a 400-fold microscope (Leica, Germany) [19].

### Wet/Dry weight ratios of lung tissues

The left lung tissue was collected and weighed, followed by drying in the oven. Then, the dry lung tissue was weighted and the Wet/Dry weight ratio was calculated.

### Enzyme-linked immunosorbent assay (ELISA)

Mouse IL-4, IL-5, and IFN-γ ELISA Kit were commercially purchased from Elabscience (E-EL-M0043c, E-EL-M0722c, E-EL-M0048c, Wuhan, China). After seeding the antibody in the well for 12 hours, the reagent was replaced with 0.1 mL testing sample or standards, followed by incubation at 37°C for 60 minutes. Then, 0.1 mL of fresh horseradish peroxidase (HRP)-conjugated antibody was introduced into each reaction well, followed by a 60-minute incubation period. 0.1 mL of the temporarily prepared 3,3′,5,5′-Tetramethylbenzidine (TMB) reagent was then introduced and cultured for 30 minutes before introducing sulfuric acid to end the reaction. The optical density at a wavelength of 450 nm was measured using a microplate reader (DeTie, China).

### Assessment of lung function

Mice in each group were incubated under anesthesia, and the parameters of lung function were measured using a Spirometer (Beijing GYD Labtech Co., Ltd., China), including PEF, RAW, and Cdyn levels.

### Oxidative stress (OS) biomarkers detection

The superoxide dismutase (SOD) activity in lung tissues was detected by utilizing an EnzyChrom Superoxide Dismutase Assay Kit (BioAssay Systems, USA) in line with the kit instructions. The reduced glutathione (GSH) level in lung tissues was determined using a commercial kit (Solarbio, China) following the kit instructions.

### Western blot analysis

Cells were loaded in the cell dish and cultured for 24 hours, followed by adding the cell lysate to be cultured at 4°C for 30 minutes. Then, cells were centrifuged at 12000× rpm for 10 minutes to obtain protein which was quantified with the bicinchoninic acid (BCA) method. The proteins were then separated using the sodium dodecyl sulfate-polyacrylamide gel electrophoresis (SDS-PAGE) and transferred to a polyvinylidene fluoride (PVDF) membrane. After blocking with 5% skim milk, p-p38 antibody (1:1000, Sino Biological, China), p-NF-κB p65 antibody (1:500, Sino Biological, China), or β-actin antibody (1:5000, Sino Biological, China) were added at 4°C overnight. Then the secondary antibodies (1:2000, Sino Biological, China) were introduced for one hour. Enhanced chemiluminescence (ECL) solution was utilized for the exposure of the bands and the ImageJ software was used for gray-level analysis [20].

### Statistical analysis

Data were listed as mean ± standard deviation (S.D.), which were analyzed using the one-way analysis of variance (ANOVA) method, followed by Tukey’s post-hoc test. The software GraphPad Prism 4.0 (GraphPad Software, USA) was used for statistical analysis. A *p*-value < 0.05 was considered statistically significant.

### Data availability

All data for this study are available on reasonable request from the corresponding author.

## RESULTS

### Arctiin attenuated OVA-challenged lung pathological alterations

In the Control and Arctiin group, the lung tissue structure was clear, with no pathological changes observed, and a thin collagen layer of the bronchial wall. In the OVA group, the lung tissue structure was evidently destroyed, with a disordered alveolar wall structure, thickened alveolar septum, reduced alveolar cavity, and significant inflammatory infiltration. The pathological state in the OVA+ Arctiin group was partly alleviated, with reduced infiltration of inflammatory cells and complete lung tissue structure (Figure 1B).

### Arctiin reduced the number of inflammatory cells in the BALF of OVA-challenged mice

**Figure 2:**
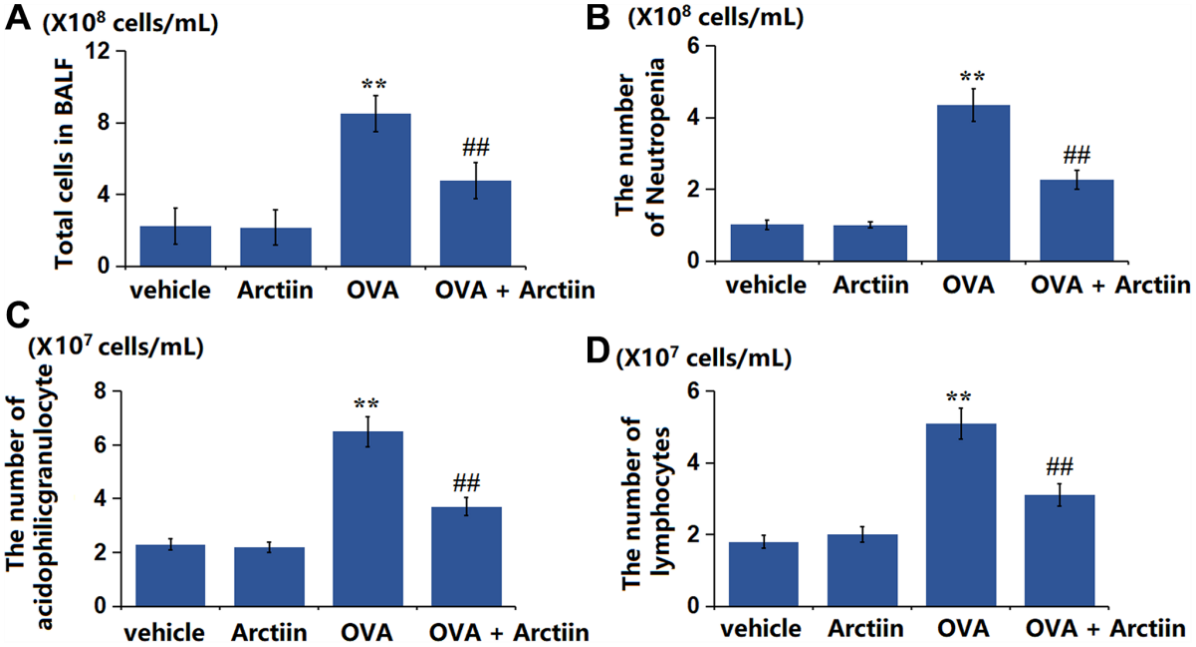
**Arctiin reduced the number of inflammatory cells in the BALF of OVA-challenged mice.** C57BL/6 mice were divided into four groups: vehicle, Arctiin, OVA, and OVA+ Arctiin (10 mg/kg). (**A**) Total cells in BALF; (**B**) The number of Neutropenia; (**C**) The number of acidophilic granulocytes; (**D**) The number of lymphocytes (*n* = 12, ^**^P < 0.01 vs. vehicle group; ^##^*P* < 0.01 vs. OVA group).

The number of BALF cells was slightly changed from 2.23 × 10^8^ cells/mL to 2.16 × 10^8^ cells/mL in the Arctiin group, was significantly increased to 4.76 × 10^8^ cells/mL in the OVA group, then signally decreased to 2.27 × 10^8^ cells/mL in the OVA+ Arctiin group (Figure 2A). Furthermore, the number of neutrophils (Figure 2B) in the control, Arctiin, OVA, and OVA+ Arctiin groups was 1.02, 1.01, 4.35, and 2.27 × 10^8^ cells/mL, respectively. The number of acidophilic granulocytes was slightly changed from 2.3 × 10^7^ cells/mL to 2.2 × 10^7^ cells/mL in the Arctiin group, was markedly increased to 6.5 × 10^7^ cells/mL in the OVA group, then observably reduced to 3.7 × 10^7^ cells/mL the OVA+ Arctiin group (Figure 2C). Moreover, the number of lymphocytes (Figure 2D) in the control, Arctiin, OVA, and OVA+ Arctiin groups was 1.8, 2, 5.1, and 3.1 × 10^7^ cells/mL, respectively. A suppressive effect of Arctiin on the number of inflammatory cells was observed in OVA-challenged mice.

### Arctiin mitigated the misbalance of Th1/Th2 factors in the lung tissues

**Figure 3:**
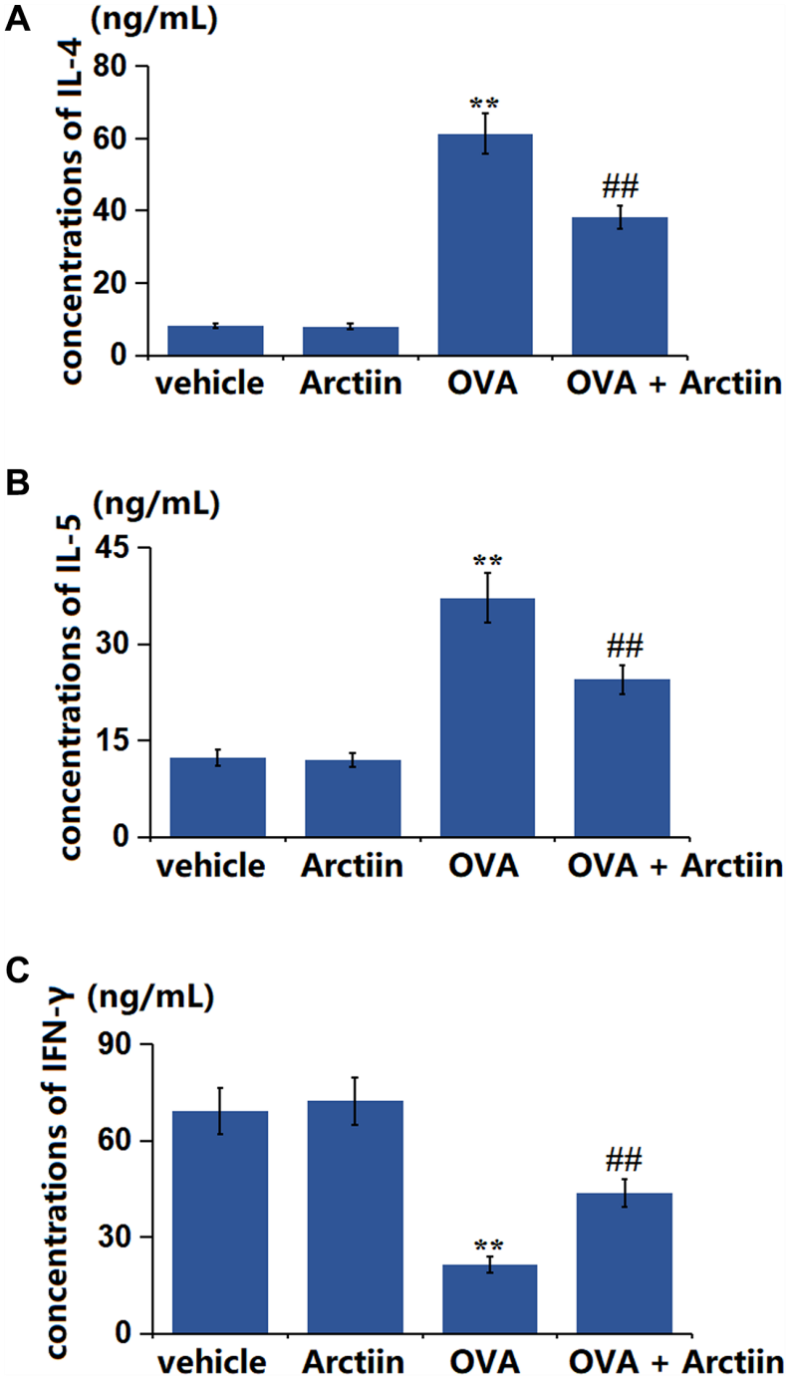
**Arctiin mitigated the disbalance of Th1/Th2 factors in the lung tissues.** C57BL/6 mice were divided into four groups: vehicle, Arctiin, OVA, and OVA+ Arctiin (10 mg/kg). (**A**) The concentrations of IL-4; (**B**) The concentrations of IL-5; (**C**) The levels of IFN-γ (*n* = 12, ^**^P < 0.01 vs. vehicle group; ^##^*P* < 0.01 vs. OVA group).

The disruption of the Th1/Th2 balance is a crucial inducer of asthma [21]. The level of IL-4 (Figure 3A) showed a slight decrease from 8.23 ng/mL to 8.01 ng/mL in the Arctiin group, a significant elevation to 61.3 ng/mL in the OVA group, and was then signally rescued to 38.2 ng/mL in the OVA+ Arctiin group. Furthermore, the levels of IL-5 in the control, Arctiin, OVA, and OVA+ Arctiin groups were 12.36, 11.95, 37.21, and 24.51 ng/mL, respectively (Figure 3B). Moreover, the IFN-γ level (Figure 3C) was marginally changed from 69.25 ng/mL to 72.32 ng/mL in the Arctiin group, was memorably decreased to 21.36 ng/mL in the OVA group; which was signally reversed to 43.6 ng/mLin the OVA+ Arctiin group.

### Arctiin ameliorated OVA-induced lung tissue impairment

**Figure 4:**
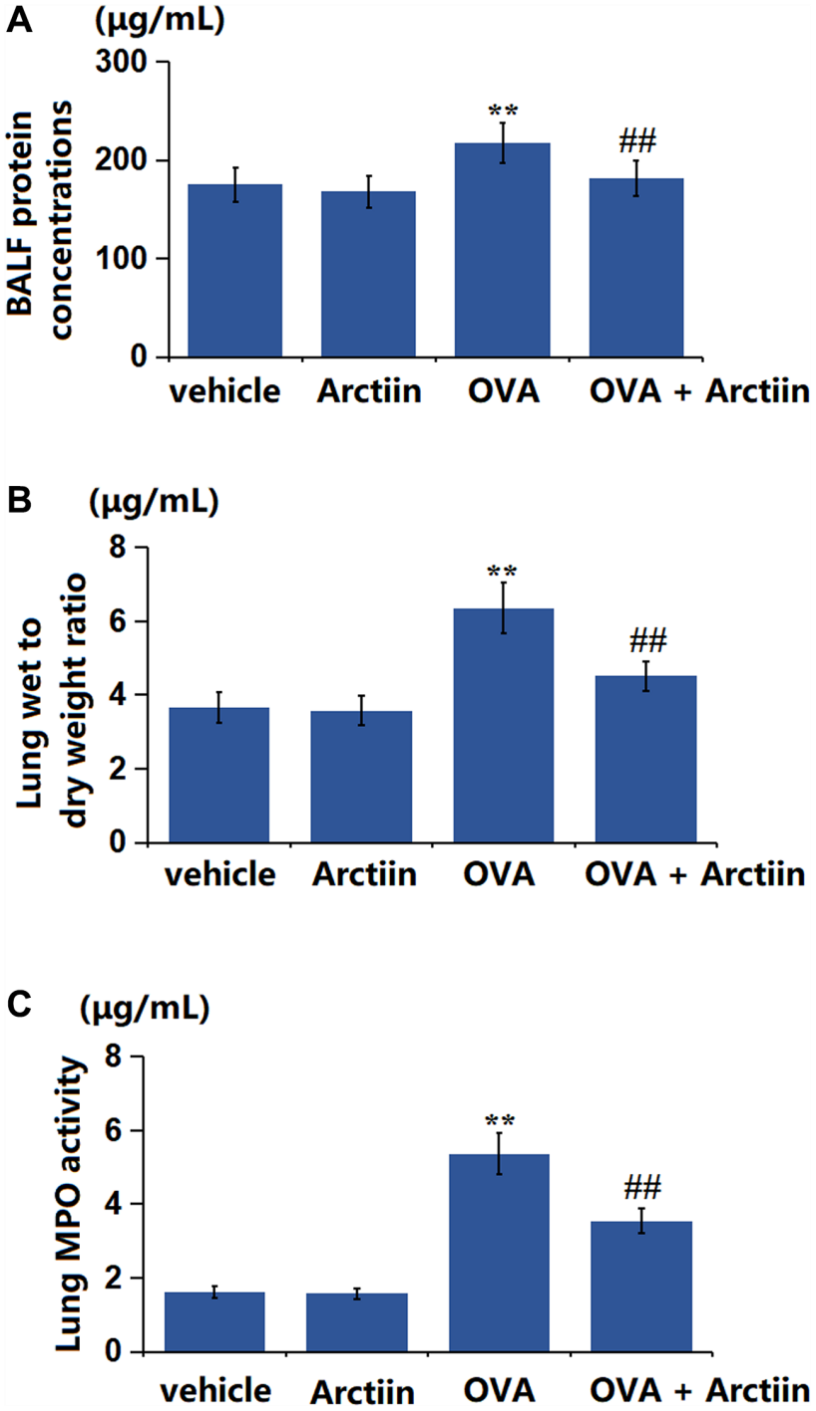
**Arctiin ameliorated OVA-induced lung tissue impairment.** C57BL/6 mice were divided into four groups: vehicle, Arctiin, OVA, and OVA+ Arctiin. (**A**) BALF protein concentrations; (**B**) Lung wet to dry weight ratio; (**C**) Lung MPO activity (*n* = 12, ^**^P < 0.01 vs. vehicle group; ^##^*P* < 0.01 vs. OVA group).

The BALF protein concentration in the control, Arctiin, OVA, and OVA+ Arctiin groups was 175.3, 168.2, 217.5, and 181.3 μg/mL, respectively (Figure 4A). Furthermore, the lung wet/dry weight ratio in the Arctiin group was slightly decreased from 3.66 to 3.58 in the Arctiin group, significantly elevated to 6.36 in the OVA group, then markedly increased to 4.51 in the OVA+ Arctiin group (Figure 4B). Additionally, the MPO activity in the control, Arctiin, OVA, and OVA+ Arctiin groups was 1.63, 1.58, 5.37, and 3.55 μg/mL, respectively (Figure 4C). A reparative effect of Arctiin on lung tissues was observed in OVA-challenged mice.

### Arctiin improved OVA-induced impairment in lung function

**Figure 5:**
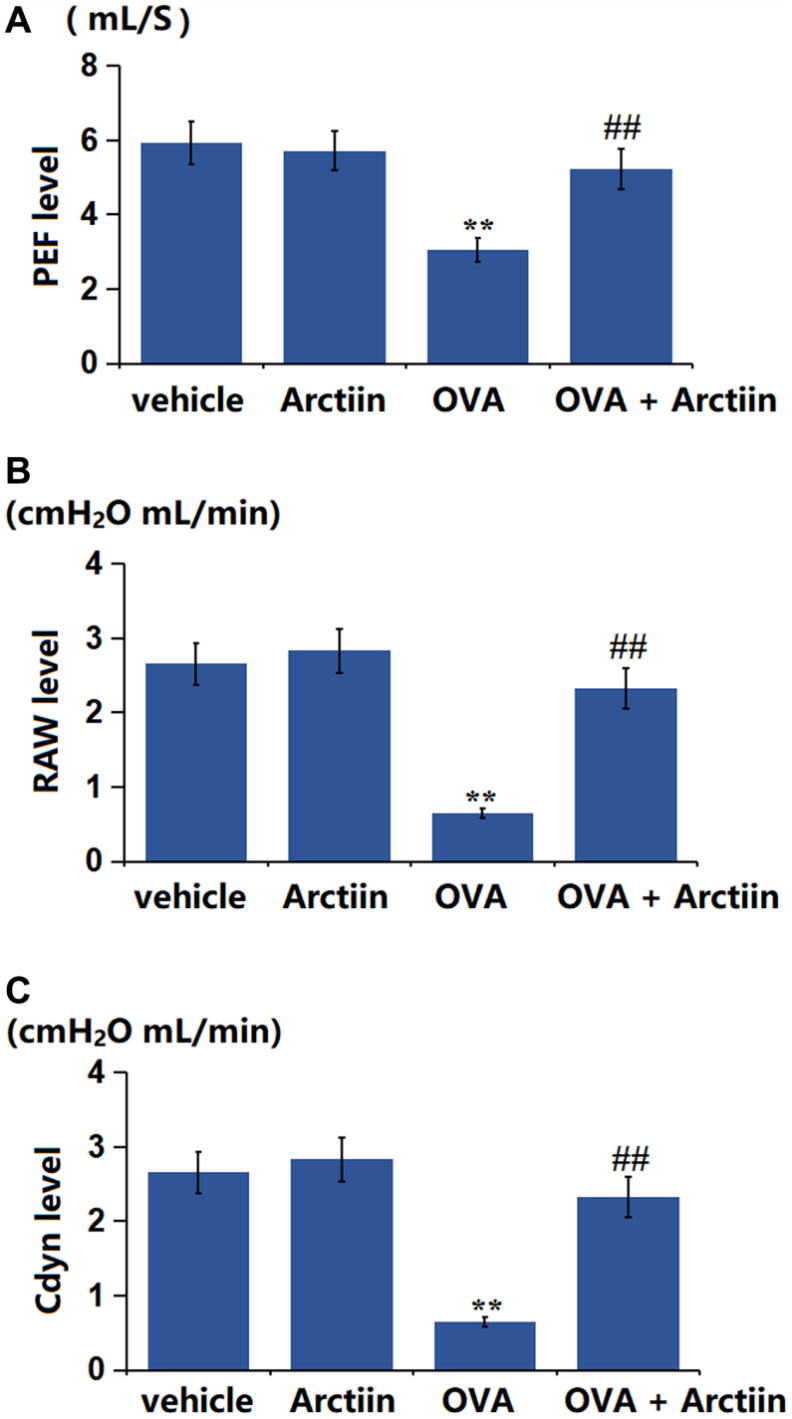
**Arctiin improved OVA-induced impairment in lung function.** C57BL/6 mice were divided into four groups: vehicle, Arctiin, OVA, and OVA+ Arctiin. (**A**) PEF; (**B**) RAW; (**C**) Cdyn level (*n* = 12, ^**^P < 0.01 vs. vehicle group; ^##^*P* < 0.01 vs. OVA group).

A Spirometer was utilized for the detection of the lung function of each animal. The PEF value in the Arctiin group decreased from 5.93 mL/s to 5.71 mL/s, was significantly dropped to 3.05 mL/s in the OVA group, then markedly restored to 5.23 mL/s in the OVA+ Arctiin group (Figure 5A). In addition, the RAW value in the control, Arctiin, OVA, and OVA+ Arctiin groups was 0.41, 0.39, 2.15, and 0.96 cmH_2_O mL/min, respectively (Figure 5B). Furthermore, the Cdyn level in the Arctiin group increased from 2.66 to 2.83 cmH_2_O mL/min and significantly reduced to 0.65 cmH_2_O mL/min in the OVA group, which was markedly reversed to 2.32 cmH_2_O mL/min in the OVA+ Arctiin group (Figure 5C). A reparative effect of Arctiin on lung function was observed in OVA-challenged mice.

### Arctiin improved OVA-induced OS in lung tissue

**Figure 6:**
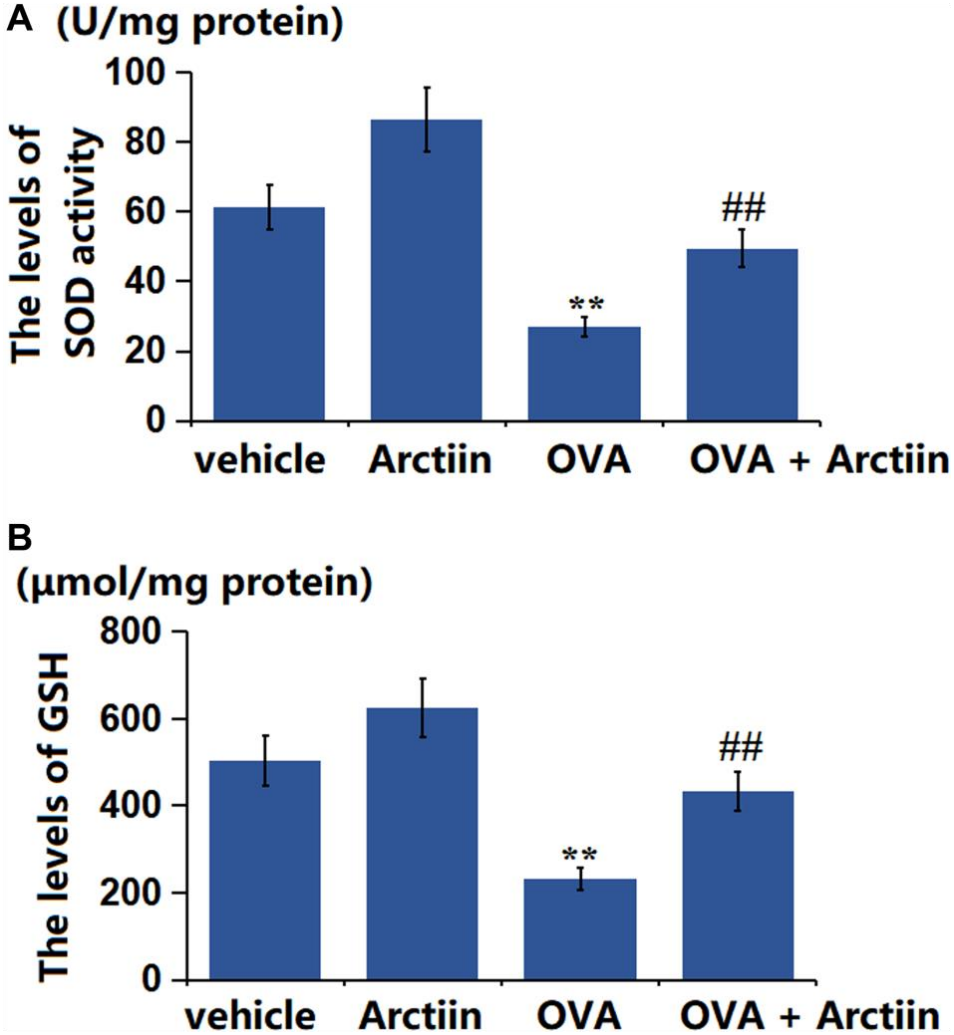
**Arctiin improved OVA-induced oxidative stress in lung tissues.** C57BL/6 mice were divided into four groups: vehicle, Arctiin, OVA, and OVA+ Arctiin. (**A**) The levels of SOD activity; (**B**) The levels of GSH (*n* = 12, ^**^P < 0.01 vs. vehicle group; ^##^*P* < 0.01 vs. OVA group).

The SOD activity was found greatly increased from 61.3 U/mg protein to 86.6 U/mg protein in the Arctiin group and significantly decreased to 26.9 U/mg protein in the OVA group, which was markedly reversed to 49.5 U/mg protein in the OVA+ Arctiin group (Figure 6A). Moreover, the GSH level in the control, Arctiin, OVA, and OVA+ Arctiin groups was 503.6, 623.8, 232.5, and 433.7 μmol/mg protein, respectively (Figure 6B). A suppressive property of Arctiin on OS in the lung tissues was observed in OVA-challenged mice.

### Arctiin prevented OVA-induced activation of p38 and NF-κB p65 in lung tissue

**Figure 7:**
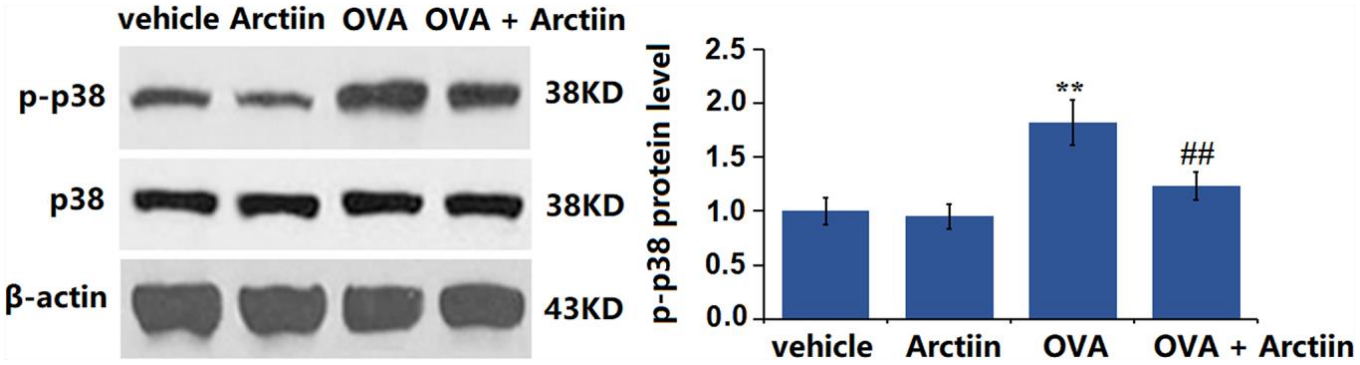
**Arctiin prevented OVA-induced activation of p38 in lung tissues.** C57BL/6 mice were divided into four groups: vehicle, Arctiin, OVA, and OVA+ Arctiin. The levels of p-p38 and total p38 were measured using western blot analysis (*n* = 12, ^**^P < 0.01 vs. vehicle group; ^##^*P* < 0.01 vs. OVA group).

**Figure 8:**
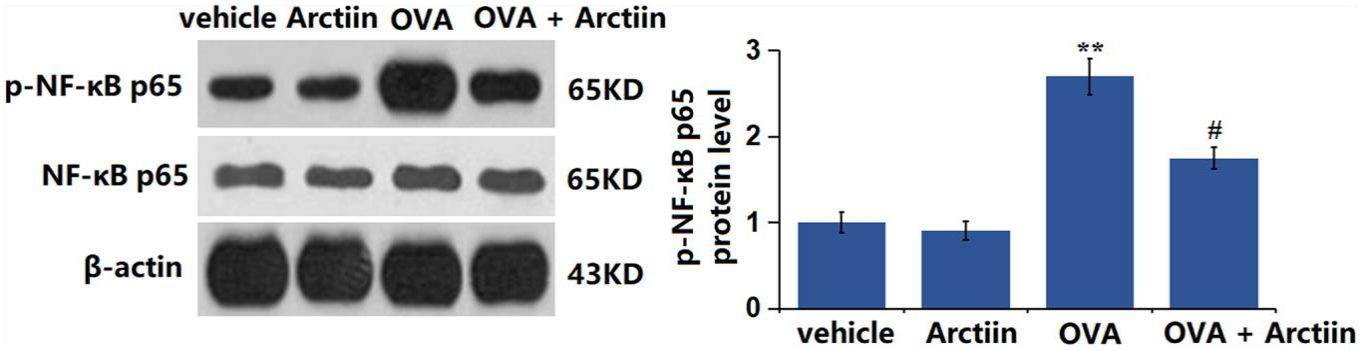
**Arctiin prevented OVA-induced activation of NF-κB p65 in lung tissues.** C57BL/6 mice were divided into four groups: vehicle, Arctiin, OVA, and OVA+ Arctiin. The levels of p-NF-κB p65 were measured using western blot analysis (*n* = 12, ^**^P < 0.01 vs. vehicle group; ^##^*P* < 0.01 vs. OVA group).

The p38 MAPK signaling and NF-κB pathway participate in the regulation of the Th1/Th2 balance [22]. The levels of p-p38/p38 (Figure 7) and p-NF-κB p65 (Figure 8) were slightly changed in the Arctiin group and significantly increased in the OVA group. However, these changes were suppressed in the OVA+ Arctiin group, implying a potential correlation between Arctiin action and p38/NF-κB signaling.

## DISCUSSION

Acidophilic granulocytes have been reported to enhance the asthmatic response, directly damage epithelial cells, increase airway responsiveness, and induce the contraction of smooth muscle by releasing LTC4, PAF, and toxic protein particles such as major basic protein (MBP), eosinophil cationic protein (ECP), and eosinophil-derived neurotoxin (EDN) [23]. In the pathogenesis of asthma, acidophilic granulocytes participate in immune regulation through the autocrine pathway and play a vital role in chronic airway inflammation by producing and secreting various bioactive protein particles [24]. Furthermore, marked neutrophil infiltration and activation are observed in asthma. Neutrophils contribute to the inflammatory response by producing LTXs, PAF, oxygen-free radicals, and lysosomal enzymes [25]. In our research on mice with an induced asthma model using OVA administration, we observed severe pathological changes, including lung histological alterations, enriched BALF proteins, increased wet/dry ratio of lung tissues, and impaired lung function. These findings were consistent with previous studies [26, 27]. However, after administering Arctiin, the pathological changes were markedly alleviated. Furthermore, consistent with Deborah’s research [28], increased infiltration of neutropenia, acidophilic granulocytes, and lymphocytes was observed in OVA-administered mice, which was notably ameliorated by Arctiin. This implies the function of Arctiin might be associated with immune regulation.

Th1 and Th2 cells are both involved in the pathogenesis and progression of asthma [29]. The classical immune theory believes that Th1/Th2 imbalance is the initiating factor for asthma [30]. A dominant Th2 response directly leads to excessive secretion of Th2 type cytokines, which aggravates airway inflammation. IL-4, mainly secreted by Th2 cells [31, 32], plays a critical role in asthma as a characteristic factor of Th2 cell subsets. IL-4 acts on B lymphocytes to produce IgE and induce a shift from IgM to IgE antibodies produced by B cells, contributing to the asthmatic response [33]. Th2 cells directly trigger the aggregation and activation of inflammatory cells by releasing IL-4, IL-5, IL-13, and other cytokines, and promote the occurrence of delayed airway allergic reactions. On the other hand, IFN-γ is a characteristic factor of the Th1 cell subset that antagonizes IL-4. It also serves as a negative growth regulator of Th2 lymphocytes and non-lymphocyte cells including epithelial cells. IFN-γ inhibits the proliferation of bronchial epithelial cells and delays airway remodeling [34]. In our research, in line with previous studies [35, 36], Th1/Th2 balance in lung tissues was found to be disrupted by OVA modeling, which was significantly rescued by Arctiin, implying a correlation between the function of Arctiin and the Th1/Th2 balance.

P38/MAPK is a member of the MAPK family, which is activated by phosphorylation when stimulated, thus participating in a variety of biological effects, such as the regulation of inflammatory response [37]. Phosphorylated p38 MAPK has been reported to phosphorylate mitogen and stress-activated protein kinase 1(MSK1) to activate NF-κB [38]. Activated NF-κB was found to regulate the expression of a variety of inflammatory molecules, such as IL-4, IL-5, IL-13, and IFN-γ, and regulate the level of Th1/ Th2 cytokines to play a role in inflammation regulation [39]. In our research, the activation of p38 MAPK/NF-κB signaling was observed in lung tissues of OVA-challenged mice, which was markedly repressed by Arctiin, suggesting that Arctiin may exert anti-asthma properties by inhibiting the p38 MAPK/NF-κB signaling pathway. In future work, we will confirm the mechanism by co-administering Arctiin and an agonist of p38 MAPK in OVA-challenged mice.

## CONCLUSION

In summary, Arctiin attenuates asthma in OVA-stimulated mice by mitigating airway inflammation and inhibiting p38 MAPK/NF-κB signaling. Therefore, the present study reveals a novel mechanism for the therapeutic potential of Arctiin in asthma.
